# Placebo-associated changes in MRI-PDFF and metabolic parameters in MASLD patients from phase Ib/IIa clinical trials: implications for early-phase trial design and translational endpoint interpretation

**DOI:** 10.3389/fphar.2026.1782232

**Published:** 2026-03-24

**Authors:** Mengdi Lu, Hong Zhang, Xiaoxue Zhu, Jia Xu, Lin Xiang, Yue Hu, Jixuan Sun, Jiajia Mai, Yanhua Ding

**Affiliations:** Phase I Clinical Research Center, First Hospital of Jilin University, Changchun, Jilin, China

**Keywords:** magnetic resonance imaging–proton density fat fraction, metabolic dysfunction–associated steatotic liver disease, metabolic parameters, phase Ib/IIa clinical trials, placebo effect, surrogate endpoint, translational trial design

## Abstract

**Background:**

The placebo effect in early-phase metabolic dysfunction–associated steatotic liver disease (MASLD) trials is poorly defined but may substantially influence endpoint interpretation and proof-of-concept decisions. A ≥30% relative reduction in magnetic resonance imaging–proton density fat fraction (MRI-PDFF) is an established noninvasive surrogate of histologic improvement. This study aimed to evaluate short-term placebo responses and 1-year longitudinal changes in hepatic fat content and metabolic parameters in phase Ib/IIa MASLD trials.

**Methods:**

A total of 41 MASLD participants from the placebo arms of five phase Ib/IIa clinical trials (2020–2024) were prospectively enrolled, with 18 completed the 1-year follow-up. Hepatic fat content (MRI-PDFF), body weight, liver enzymes, and lipid parameters were assessed from baseline to week 56.

**Results:**

After 4–5 weeks of placebo treatment, 20% of participants achieved ≥30% hepatic fat reduction, with mean absolute and relative decreases of 2.48% (SD: 2.88) and 16.47% (SD: 23.46), respectively. Body weight decreased by 2.5 kg (SD: 1.8), corresponding to a relative reduction of 2.73% (SD: 1.97). Additionally, liver enzymes and most lipid parameters declined, whereas triglycerides showed a modest increase. During 1-year follow-up, hepatic fat content and body weight rebounded from end-of-treatment levels but remained below baseline. Liver enzymes partially rebounded but stayed below baseline, while lipid parameters exceeded baseline values.

**Conclusion:**

Early-phase MASLD trials show a measurable, heterogeneous placebo effect with partial rebound after treatment cessation. These findings underscore the importance of explicitly accounting for short-term placebo-associated changes when designing early-phase trials, selecting surrogate endpoints and interpreting preliminary efficacy signals.

## 1 Introduction

Metabolic dysfunction–associated steatotic liver disease (MASLD) is one of the most common chronic liver diseases worldwide, with a prevalence of 25%–45% (Radosavljevic et al., 2024; European Association for the Study of the Liver et al., 2024a; European Association for the Study of the Liver et al., 2024b). Currently, the U.S. Food and Drug Administration (FDA) has approved resmetirom (a thyroid hormone receptor-β agonist) and semaglutide (Glucagon-like peptide-1 receptor agonist, GLP-1R) for the treatment of adult patients with metabolic dysfunction–associated steatohepatitis (MASH) and moderate-to-advanced fibrosis without cirrhosis (Harrison et al., 2019; H et al., 2024; Keam, 2024; O'Leary, 2025). While these developments have advanced the therapeutic landscape, many MASLD drug trials—especially early-phase studies—have failed. These failures are often driven by small sample sizes, substantial and variable placebo responses, and uncertainty regarding optimal surrogate endpoints and fibrosis reversal timelines, underscoring critical challenges in early-phase MASLD trial design and efficacy interpretation (Do et al., 2025).

In MASLD clinical trials, liver biopsy remains the gold standard for evaluating treatment response, typically assessed by two endpoints: (Radosavljevic et al., 2024): resolution of MASH without fibrosis worsening, and (European Association for the Study of the Liver et al., 2024a) ≥1-stage fibrosis improvement without MASH progression (H et al., 2024). However, its invasiveness limits widespread use in early-phase clinical trials (Jayakumar et al., 2019; Rockey et al., 2009). Magnetic resonance imaging–proton density fat fraction (MRI-PDFF), a highly reproducible and non-invasive modality, is increasingly used as a surrogate endpoint (Harrison et al., 2025; Beyer et al., 2025). A ≥30% relative reduction in MRI-PDFF is strongly associated with histologic improvement, supporting its use for noninvasive monitoring in early-phase MASLD clinical trials (Stine et al., 2021; Tamaki et al., 2022). Additionally, in MASH patients, weight loss of ≥5%, ≥7%, and ≥10% can reduce steatosis, resolve steatohepatitis, and improve or stabilize fibrosis, respectively (Younossi et al., 2021).

Significant improvements in histology and MRI-PDFF are often observed even in placebo groups, known as the “placebo effect” (Neuschwander-Tetri et al., 2015; Ampuero et al., 2022). Analyses of multiple II/III MASH clinical trials have shown that 10%–28% of placebo participants achieve ≥30% MRI-PDFF reduction or histological improvement (Ampuero et al., 2022; Nedrud et al., 2023; Han et al., 2019; Pennisi et al., 2022; Ng et al., 2022; Rowe and Parker, 2022). MASLD is a metabolic disease, and lifestyle factors such as diet and exercise can significantly influence hepatic fat content and metabolic parameters, thereby contributing to placebo-associated changes (Wong et al., 2018; Mucinski et al., 2024).

Early-phase MASLD clinical trials are particularly vulnerable to placebo effects because of short treatment durations, small sample sizes, and limited statistical power. In some early-phase MASLD clinical trials, placebo effect has approached those of active treatments (Xie et al., 2025; Xiang et al., 2025), potentially confounding efficacy assessments. Small sample sizes and limited statistical power in phase Ib/IIa MASLD clinical trials hinder clear understanding, affecting trial design, sample size, and endpoint selection. Systematically characterizing placebo frequency and trajectory is therefore essential to optimize early-phase trial design and accurately interpret preliminary pharmacologic signals.

Obesity, insulin resistance, type 2 diabetes mellitus (T2DM), dyslipidemia, and metabolic syndrome are key factors driving the onset and progression of MASLD (Soto et al., 2024; St et al., 2025; Younossi et al., 2025; Zargar et al., 2025). In this study, we analyzed MASLD placebo arms from multiple phase Ib/IIa clinical trials with 4–5 weeks of treatment and conducted longitudinal follow-up for up to 1 year. By evaluating changes in hepatic fat content assessed by MRI-PDFF, body weight, and key metabolic parameters (liver enzymes and lipid parameters), we aimed to delineate the short-term placebo effect and post-treatment dynamics, providing practical, data-driven guidance for early-phase MASLD drug development by informing endpoint selection, sample size estimation, and interpretation of early pharmacodynamic signals in translational clinical trials.

## 2 Methods

### 2.1 Study design

This prospective observational study enrolled participants from the placebo arms of five phase Ib/IIa MASLD, T2DM, or obesity clinical trials with 4/5 weeks treatments. After the intervention, participants were followed for 1 year. The study was conducted in accordance with the Declaration of Helsinki and approved by the Ethics Committee of the First Hospital of Jilin University (Ethical Approval Number: 19K096001; approval date: 19 September 2019). Written informed consent was obtained from all participants prior to study initiation.

### 2.2 Participants

A total of 41 placebo-treated participants were enrolled from clinical trials conducted between January 2020 and December 2024 at the Phase I Clinical Research Center of the First Hospital of Jilin University, with 18 participants completing a 1-year follow-up.

Inclusion criteria were: (Radosavljevic et al., 2024): aged 18–65 years; (European Association for the Study of the Liver et al., 2024a); stable body weight (≤5% change) within 3 months before enrollment; and (European Association for the Study of the Liver et al., 2024b) baseline MRI-PDFF >5% and meeting MASLD diagnostic criteria (Rinella et al., 2023a; Rinella et al., 2023b). Exclusion criteria included: (Radosavljevic et al., 2024): incomplete trial participation; (European Association for the Study of the Liver et al., 2024a); combination of other chronic liver diseases (hepatitis B or C, autoimmune hepatitis, hemochromatosis, Wilson’s disease, and α1-antitrypsin deficiency) or alcohol-related liver disease; (European Association for the Study of the Liver et al., 2024b); decompensated cirrhosis; (Harrison et al., 2019); history of bariatric surgery; (H et al., 2024); type 1 diabetes; and (Keam, 2024) current use of medications affecting hepatic steatosis.

All clinical trials were registered on ClinicalTrials.gov or the China Clinical Trial Registry (https://www.chictr.org.cn), and all participants met the respective trial inclusion and exclusion criteria. The cohort included participants from three published clinical trials—NCT04140123 (tumor necrosis factor α pathway regulators ZSP1601) with seven of nine placebo participants (two were excluded due to missing data) (Hu et al., 2023), NCT05943886 (glucagon-like peptide-1/fibroblast growth factor 21 dual agonist HEC88473) with seven of 10 placebo participants (three participants were excluded: two because their baseline MRI-PDFF was ≤5%, and one due to early study discontinuation related to an adverse event) (Xiang et al., 2025) and NCT06491576 (adenosine triphosphate–citrate lyase inhibitor BGT-002) with 12 placebo participants (Xie et al., 2025)—and two unpublished clinical trials—NCT06778850 (glucagon-like peptide-1 receptor agonist MDR-001) with six of eight placebo participants (two participants were excluded for having baseline MRI-PDFF ≤5%) and CTR20220345 (gut-liver anti-inflammatory metabolic modulator, berberine ursodeoxycholate HTD1801) with nine of 12 placebo participants (three participants were excluded for having baseline MRI-PDFF ≤5%).

During both the treatment and follow-up periods, participants continued concomitant medications and maintained their usual diet and physical activity levels.

### 2.3 Data collection

Baseline demographic data collected included age, gender, ethnicity, body weight, body mass index (BMI). Metabolic parameters included hemoglobin A1c (HbA1c), fasting blood glucose (FBG), fasting insulin, alanine aminotransferase (ALT), aspartate aminotransferase (AST), gamma-glutamyl transferase (GGT), alkaline phosphatase (ALP), total cholesterol (TC), triglycerides (TG), low-density lipoprotein (LDL), and high-density lipoprotein (HDL). Imaging parameters included hepatic fat content (MRI-PDFF), controlled attenuation parameter (CAP), and liver stiffness (LSM) by transient elastography. Missing post-trial data (weight, CAP, LSM) were due to trial design. Data were collected at baseline (week 0), end of trial (week 4/5), and 1-year follow-up (week 56).

### 2.4 MRI-PDFF technique

All participants underwent abdominal MRI using the same GE Discovery 750 3.0T system. MRI-PDFF enables precise quantification of hepatic water and fat content through iterative decomposition using asymmetric echoes and least-squares estimation (IDEAL IQ, GE Discovery 750 3.0T). Cross-sectional IDEAL IQ imaging encompassed the entire liver. Key imaging parameters were: acquisition matrix = 256 × 256, echo time (TE) = 3 ms, repetition time (TR) = 6 ms, flip angle = 3°, field of view (FOV) = 480 mm, slice thickness = 9 mm, and a single breath-hold acquisition time of 19 s. On single-slice images of the right posterior, right anterior, left lateral, and left medial liver segments, regions of interest (ROIs) of 284 mm^2^ were manually placed, carefully avoiding focal liver lesions, large vessels, artifacts, and bile ducts. The fat fraction was measured in each of the four ROIs, and the mean value was calculated to represent the overall hepatic fat fraction.

### 2.5 Outcome measures

The primary endpoint was a ≥30% relative reduction in MRI-PDFF after 4/5 weeks of placebo, a threshold closely linked to histological improvement in MASH (Stine et al., 2021). Secondary efficacy endpoints included: (a) MRI-PDFF normalization (≤5%), (b) ≥30% relative increase in MRI-PDFF (Nedrud et al., 2023), and (c) ≥5% relative body weight loss (Younossi et al., 2021).

Additionally, the study assessed dynamic changes in MRI-PDFF, body weight, BMI, liver enzymes (ALT, AST, GGT, ALP), and lipid parameters (TC, TG, LDL). Two observation periods were defined: Period 1, baseline to trial end (week 0–4/5; n = 41), and Period 2, trial end to 1-year follow-up (week 4/5–56; n = 18). A stratified analysis was conducted (baseline to week 4/5, baseline to week 56, and week 4/5 to week 56) to assess longitudinal trends and to determine whether these measures returned to baseline at week 56.

### 2.6 Statistical analysis

Descriptive analyses were performed for all 41 placebo-treated MASLD participants and for the 18 participants who completed the 1-year follow-up. Continuous variables are reported as mean ± SD, and categorical variables as counts (n) and percentages (%). Normality of continuous variables was assessed using the Shapiro–Wilk test. Pre- and post-treatment comparisons used paired t-tests if normally distributed or Wilcoxon signed-rank tests otherwise. Baseline differences and changes among trials were analyzed using Kruskal–Wallis H test with Dunn’s *post hoc* or One-way Analysis of Variance (ANOVA) with Tukey’s Honestly Significant Difference (HSD) for continuous variables, and χ^2^ test for categorical variables. Analyses were conducted in R software (version 4.4.2) and GraphPad Prism (version 10.0), with P < 0.05 considered statistically significant.

## 3 Results

### 3.1 Participant baseline characteristics

This study included a total of 41 MASLD participants who completed 4/5 weeks of placebo treatment. The mean age was 41.0 ± 11.0 years, with 16 females (39%). Mean body weight and BMI were 81.5 ± 14.0 kg and 29.4 ± 4.5 kg/m^2^, respectively. Type 2 diabetes was present in 51% (21/41), with mean HbA1c of 7.4% ± 1.4%. Baseline MRI-PDFF, CAP, and LSM averaged 14.56% ± 8.60%, 314.5 ± 43.9 dB/m, and 7.32 ± 2.93 kPa, respectively, and mean ALT was 53.29 ± 35.91 U/L (Supplementary Table S1).

No significant baseline differences were observed across trials for age, gender, HOMA-IR, ALP, TC, TG, LDL, and HDL. However, significant differences were found for body weight, BMI, HbA1c, FBG, ALT, AST, GGT, MRI-PDFF, CAP, and LSM (Supplementary Table S1).

After the 4/5 weeks placebo treatment, trial one and trial four differed significantly in absolute MRI-PDFF change (P = 0.003), while other groups showed no significant differences (Supplementary Figure S1A). Relative MRI-PDFF changes were no clear statistically significant differences across trials (Supplementary Figure S1B). In four of the five trials (excluding trial 2), a ≥30% MRI-PDFF reductions were observed, indicating a placebo effect (Supplementary Figure S1B).

### 3.2 Changes in hepatic fat content following placebo treatment

As shown in Table 1, participants in the placebo group exhibited a significant reduction in MRI-PDFF at the end of the 4/5 weeks clinical trials (P < 0.0001). The mean absolute reduction in MRI-PDFF was 2.48% (SD: 2.88), corresponding to a relative reduction of 16.47% (SD: 23.46). Overall, 20% (8 of 41) of participants achieved a relative reduction in MRI-PDFF of ≥30%, while 2% (1 of 41) experienced a relative increase of ≥30%. Additionally, 20% (8 of 41) of participants reached a normalized MRI-PDFF level of ≤5% (Table 2).

**TABLE 1:** Pre- and post-treatment changes in placebo-treated patients across the five parent trials.

Characteristic	Pre-treatment	Post-treatment	Mean change	Mean change percent	P value	N
Body weight, kg	85.9 (12.2)	83.4 (11.2)	−2.5 (1.8)	−2.73 (1.97)	<0.0001	32
BMI, kg/m^2^	30.6 (4.2)	29.7 (3.8)	−0.9 (0.6)	−2.73 (1.97)	<0.0001	32
HbA1c, %	7.8 (1.5)	7.6 (1.5)	−0.2 (0.6)	−2.59 (7.20)	0.1489	22
FBG, mmol/L	7.44 (2.42)	7.48 (3.32)	0.03 (2.37)	0.43 (25.32)	0.1663	41
HOMA-IR	5.3 (3.0)	5.2 (3.2)	−0.1 (2.5)	5.44 (49.18)	0.8355	29
ALT, U/L	53.29 (35.91)	49.34 (31.21)	−3.95 (18.41)	−1.52 (34.92)	0.1663	41
AST, U/L	36.84 (25.29)	34.05 (23.44)	−2.79 (18.61)	−2.98 (33.84)	0.0286	41
GGT, U/L	73.80 (58.10)	62.82 (56.28)	−10.97 (28.07)	−15.54 (25.15)	<0.0001	41
ALP, U/L	87.51 (21.15)	85.12 (22.63)	−2.40 (13.38)	−2.11 (15.68)	0.3267	41
TC, mmol/L	5.31 (0.86)	5.03 (0.87)	−0.28 (0.73)	−4.49 (14.25)	0.0187	41
TG, mmol/L	2.69 (1.13)	2.82 (1.88)	0.13 (1.63)	8.54 (60.96)	0.391	41
LDL, mmol/L	3.41 (0.67)	3.22 (0.69)	−0.19 (0.49)	−4.94 (14.86)	0.0154	41
HDL, mmol/L	1.16 (0.24)	1.05 (0.17)	−0.11 (0.17)	−7.97 (14.37)	<0.0001	41
MRI-PDFF, %	14.56 (8.60)	12.08 (7.80)	−2.48 (2.88)	−16.47 (23.46)	<0.0001	41
CAP, db/m	314.5 (43.9)	309.5 (49.9)	−5.0 (36.7)	−1.31 (11.75)	0.4969	26
LSM, kPa	7.32 (2.97)	6.51 (1.91)	−0.80 (2.53)	−4.35 (32.64)	0.0613	26

Data are presented as mean (standard deviation). N, the number of participants.

**TABLE 2:** Observed relative changes in hepatic fat content and body weight following 4/5 weeks placebo treatment.

Subjects	MASLD (N)	≥30% relative increase in MRI-PDFF	≥30% relative reduction in MRI-PDFF	MRI-PDFF normalization (≤5% at end of study)	≥5% relative reduction in body weight	≥7% relative reduction in body weight
Trial 1	12	0	3 (25%)	0	0	0
Trial 2	7	0	0	0	0	0
Trial 3	7	0	2 (29%)	2 (29%)	0	0
Trial 4	9	1 (11%)	1 (11%)	3 (33%)	NA	NA
Trial 5	6	0	2 (33%)	3 (50%)	3 (50%)	0
Total	41	1/41 (2%)	8/41 (20%)	8/41 (20%)	3/32 (9%)	0

Data are presented as number (percent). N, the number of participants; NA, not available; MRI-PDFF, magnetic resonance imaging–proton density fat fraction; MASLD, metabolic dysfunction–associated steatotic liver disease.

### 3.3 Changes in body weight and BMI following placebo treatment

Compared with baseline, both body weight and BMI showed significant decreases by the end of the 4/5 weeks placebo treatment period (P < 0.0001). The mean reductions were 2.5 kg (SD: 1.8) for body weight and 0.9 kg/m^2^ (SD: 0.6) for BMI. These corresponded to identical relative reductions of 2.73% (SD: 1.97) for both parameters (Table 1). Among the participants, 9% (3/32) achieved a ≥5% reduction in body weight, and none achieved a reduction greater than 7% (Table 2). Notably, 67% (2/3) of participants who lost 5% to <7% of their baseline body weight also achieved a ≥30% reduction in MRI-PDFF.

### 3.4 Changes in liver enzymes and lipid parameters following placebo treatment

Compared with baseline, significant reductions were observed in AST and GGT levels, while changes in ALT and ALP were not statistically significant at end of the 4/5 weeks placebo treatment. Specifically, ALT and ALP decreased by 3.95 U/L (SD: 18.41; P = 0.1663) and 2.40 U/L (SD: 13.38; P = 0.3267), respectively. In contrast, AST and GGT decreased by 2.79 U/L (SD: 18.61; P = 0.0286) and 10.97 U/L (SD: 28.07; P < 0.0001), respectively (Table 1).

Regarding lipid parameters, TC and LDL significantly decreased by 0.28 mmol/L (SD: 0.73; P = 0.0187) and 0.19 mmol/L (SD: 0.49; P = 0.0154), respectively. TG showed a slight but non-significant increase of 0.13 mmol/L (SD: 1.63; P = 0.391) (Table 1).

### 3.5 Changes in hepatic fat content during 1-year follow-up

**FIGURE 1:**
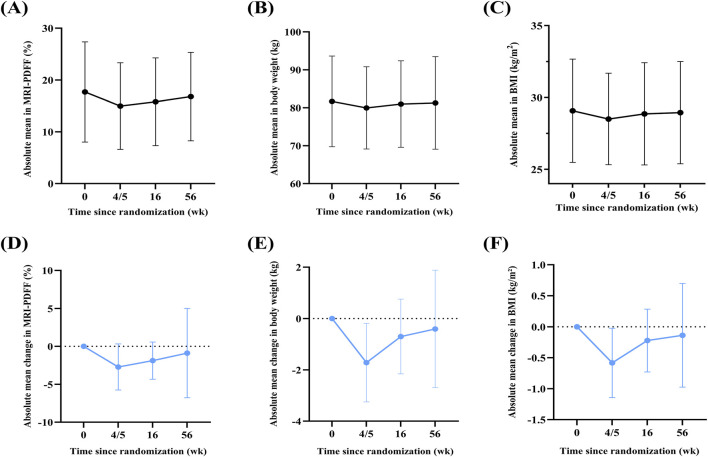
Absolute mean values and mean changes in hepatic fat content, body weight, and body mass index (BMI) among time points. **(A–C)** Absolute mean values of MRI-PDFF **(A)**, body weight **(B)**, and BMI **(C)** at weeks 0, 4/5, 16, and 56. **(D–F)** Mean changes from baseline in MRI-PDFF **(D)**, body weight **(E)**, and BMI **(F)** at week 4/5, 16, and 56, compared to week 0, respectively. Data plotted as mean or mean changes ±standard deviation (SD); The X-axis represents time since randomization (week), with key evaluation points at week 0 (baseline), week 4/5 (end of trial treatment), week 16 (3-month follow-up), and week 56 (1-year follow-up).

18 participants were followed for 1 year after the 4/5 weeks placebo treatment. MRI-PDFF rose from week 4/5 to week 56 but stayed below baseline (Supplementary Table S2; Figures 1A,D). At week 56, MRI-PDFF increased by 1.83% (SD: 5.93) from week 4/5, corresponding to relative increases of 18.91% (SD: 54.53). Compared to baseline, MRI-PDFF decreased by 0.89% (SD: 5.89) at week 56, corresponding to a relative reduction of 1.58% (SD: 35.50) (Supplementary Tables S3–S4).

A significant placebo effect (≥30% reduction in MRI-PDFF from baseline) was observed in 17% of participants at week 4/5 and week 16, and in 22% at week 56, suggesting a sustained effect throughout follow-up (Supplementary Figure S2A). Additionally, 11% (2 of 18) of participants had a further ≥30% decrease in MRI-PDFF from week 4/5 to week 56, while another 22% (4 of 18) showed a ≥30% increase (Supplementary Figure S2B).

### 3.6 Changes in body weight and BMI during 1-year follow-up

Body weight and BMI rose from week 4/5 to week 56 but stayed below baseline (Supplementary Table S2; Figures 1B,C,E,F). At week 56, body weight and BMI increased by 1.3 kg (SD: 3.0) and 0.5 kg/m^2^ (SD: 1.1) (Supplementary Table S3), corresponding to relative increases of 1.48% (SD: 3.74) and 1.45% (SD: 3.77), respectively (Supplementary Table S4). Compared to baseline, body weight and BMI were slightly lower at week 56 by 0.4 kg (SD: 2.3) and 0.1 kg/m^2^ (SD: 0.8), with mean relative reductions of 0.53% (SD: 2.72) and 0.51% (SD: 2.76), respectively (Supplementary Tables S3–S4).

No participants lost ≥5% of body weight at week 4/5 or 16; however, 6% (1/18) experienced a 5% to <7% reduction by week 56 (Supplementary Figure S2C). After 1 year, compared with week 4/5, 6% (1/18) experienced a 5% to <7% loss, whereas 22% (4/18) gained 5% to <7% of body weight (Supplementary Figure S2D).

### 3.7 Changes in liver enzymes and lipid parameters during 1-year follow-up

**FIGURE 2:**
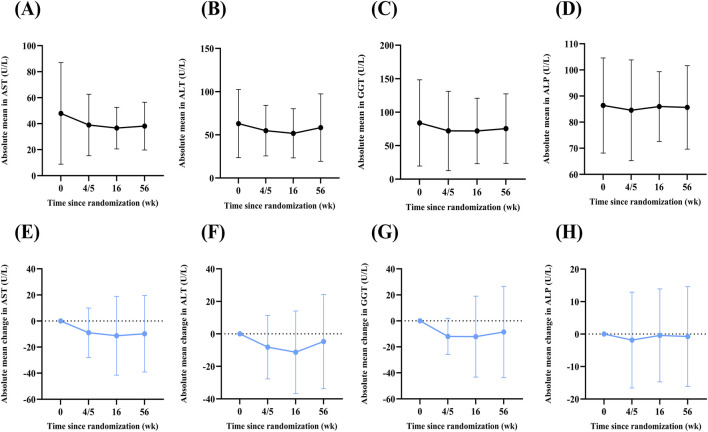
Absolute mean values and mean changes in liver enzymes across time points. **(A–D)** Absolute mean values of AST **(A)**, ALT **(B)**, GGT **(C)**, and ALP **(D)** at weeks 0, 4/5, 16, and 56 **(E–H)** Mean changes in AST **(E)**, ALT **(F)**, GGT **(G)**, and ALP **(H)** from baseline at week 4/5, 16, and 56. Data are presented as mean or mean change ±standard deviation (SD).

During the 1-year follow-up, AST continued to decline, whereas ALT, GGT, and ALP showed partial rebounds but remained below baseline (Supplementary Table S2; Figure 2). By week 56, AST had decreased by 0.84 U/L (SD: 19.36) from week 4/5, while ALT, GGT, and ALP increased by 3.48 U/L (SD: 24.41), 3.42 U/L (SD: 38.22), and 1.09 U/L (SD: 15.13), respectively (Supplementary Table S3; Figure 2).

Regarding lipid parameters, by week 56, all three (TC, TG, LDL) slightly exceeded base-line but fluctuated within a narrow range (Supplementary Table S2; Supplementary Figure S3). Mean increases were 0.29 mmol/L (SD: 0.71) for TC, 0.09 mmol/L (SD: 2.19) for TG, and 0.18 mmol/L (SD: 0.58) for LDL from week 4/5 to week 56 (Supplementary Table S3; Supplementary Figure S3).

## 4 Discussion

Few studies have tracked long-term changes in hepatic fat content, body weight, liver enzymes, and lipid parameters in placebo-treated MASLD participants in short-duration phase Ib/IIa clinical trials. To our knowledge, this is the first study to assess placebo effect in such populations. To our knowledge, this study represents the first integrated analysis focusing specifically on the placebo effect in this early-phase setting. By pooling MASLD placebo groups participants from five phase Ib/IIa trials with treatment durations of only 4–5 weeks, we demonstrate that clinically meaningful placebo-associated reductions in hepatic steatosis can occur rapidly and persist beyond treatment discontinuation.

In our cohort, 20% of participants showed a ≥30% relative reduction in MRI-PDFF after placebo treatment, with a mean absolute reduction of 2.48% and mean relative reduction of 16.47%. These results are consistent with a study of 187 placebo participants, which reported ≥30% relative reductions in 20% and 28% of participants at 12 and 24 weeks, respectively, with an estimated absolute reduction of 2.3% and median relative reduction of 13% at 24 weeks (Nedrud et al., 2023). The consistency of effect size across markedly different trial durations underscores that placebo-associated changes in hepatic fat content can emerge early, even within weeks of trial initiation. This observation has important implications for early-phase MASLD drug development, where short treatment durations and limited sample sizes are common.

A meta-analysis of 39 MASH randomized controlled trials (RCTs) (1,463 placebo participants, ≥22 weeks) reported mean reductions of 2.43% in MRI-PDFF, 0.28 kg/m^2^ in BMI, and significant decreases in ALT and AST (Han et al., 2019). Another meta-analysis of 2,649 placebo-treated MASLD patients found Asians had the largest BMI drop (0.84 kg/m^2^), with shorter trials showing greater reductions; Mean changes in LDL, HDL, and TG were −0.18, −0.009, and −0.11 mmol/L, respectively (Ng et al., 2022). In our study, after 4/5 weeks of placebo, 41 participants experienced significant reductions in body weight (−2.5 ± 1.8 kg), BMI (−0.9 ± 0.6 kg/m2), and MRI-PDFF (−2.48% ± 2.88%). Liver enzymes (AST, ALT, ALP) trended downward, LDL and HDL decreased slightly, and TG increased modestly. Overall, the trends were consistent with published data, with minor differences likely due to sample size, ethnicity, trial duration, and the limited responsiveness of lipid parameters to placebo.

MASLD is a chronic metabolic disease with alternating remission and progression (Mejía-Guzmán et al., 2025; Loomba and Adams, 2019). A meta-analysis of 43 RCTs with 2,649 placebo-treated MASLD patients (mostly ≥6 months) reported MASH resolution in 11.7%, NAS improvement ≥2 points without fibrosis worsening in 21%, and ≥1-stage fibrosis improvement in 18.8%, while fibrosis progressed in 22% (Ng et al., 2022). In our study, 18 placebo-treated participants were followed for 56 weeks. The proportion achieving ≥30% relative MRI-PDFF reduction was 17% at weeks 4/5 and 16, rising to 22% at week 56. Between weeks 4/5 and 56, 11% (2/18) newly reached ≥30% reduction, while 22% (4/18) experienced increases ≥30%. Overall, the magnitude of these fluctuations in early-phase clinical trials is comparable to histological MASH remission rates in later-phase (IIb/III) MASLD clinical trials. These results show that placebo effect can appear early and fluctuate over time. Notably, the magnitude of these early placebo-associated changes overlaps with effect sizes commonly used to support proof-of-concept decisions in phase Ib/IIa MASLD trials, indicating that placebo responses represent a critical translational signal rather than random variability.

Changes in MRI-PDFF among placebo-treated participants may be related to metabolic risk factors such as T2DM and obesity (Ng et al., 2022). Although baseline HbA1c, FBG, BMI, body weight, and MRI-PDFF varied across the five trials, the mean relative change in MRI-PDFF after 4/5 weeks of placebo treatment was not statistically significant (P > 0.05); therefore, the data were pooled to assess the early-phase placebo effect. Baseline MRI-PDFF was higher in participants without T2DM than with T2DM (P < 0.01), but absolute and relative changes post-placebo did not differ between trials (P > 0.05).

Previous studies suggest that the Hawthorne effect—behavioral changes resulting from observation—may improve adherence to diet and exercise in randomized controlled trials (Pennisi et al., 2022; McCarney et al., 2007; Abraham et al., 2018; Glass et al., 2020). Given MASLD’s metabolic nature, lifestyle changes can slow disease progression and reduce hepatic fat and enzymes (Romero-Gómez et al., 2017) (Keating and Adams, 2016). In this analysis, placebo-associated changes persisted over 1 year, with some participants showing further reduction while others experienced increases in MRI-PDFF. Early placebo responses may also be influenced by adverse events, administration route, inpatient stay, and baseline disease severity—for example, nausea in the treatment group may indirectly cause weight loss in placebo-treated participants. These findings indicate that placebo effect is multifactorial and should be carefully considered when evaluating drug efficacy in phase Ib/IIa MASLD clinical trials, especially when treatment–placebo differences are small.

From a translational pharmacology perspective, these findings underscore the importance of explicitly accounting for placebo-associated changes when designing and interpreting phase Ib/IIa MASLD trials. Failure to consider such effects may obscure true pharmacologic signals, particularly when treatment–placebo differences are modest. Accordingly, the use of appropriate control arms, robust endpoint selection, and consideration of longitudinal placebo trajectories are essential for accurate efficacy assessment and dose selection in early-stage MASLD drug development. Collectively, these results provide important translational pharmacology insights, facilitating interpretation of short-term pharmacodynamic signals and reducing the risk of false-positive proof-of-concept conclusions.

This study has several limitations: histological data were not available, precluding direct assessment of histological placebo effect; the pooled analysis included a relatively small sample size from a single center; heterogeneity in trial inclusion criteria may have influenced effect estimates; and specifically, variability in lifestyle adherence may have contributed to heterogeneity in MRI-PDFF and metabolic outcomes. Nonetheless, the consistency of our findings with larger external datasets supports their generalizability. Future multi-center studies with integrated imaging and histological assessments are warranted to further refine the interpretation of placebo effects in early-phase MASLD clinical trials.

## 5 Conclusion

Approximately 20% of placebo-treated MASLD participants achieved a ≥30% reduction in MRI-PDFF within 4–5 weeks, accompanied by modest improvements in body weight and metabolic parameters, with partial rebound over 1 year. These findings demonstrate that short-term placebo-associated changes can reach clinically meaningful thresholds commonly used in early drug development. Explicitly accounting for these effects is therefore essential for endpoint selection, sample size estimation, and accurate interpretation of pharmacodynamic signals in phase Ib/IIa MASLD trials.

## Data Availability

The original contributions presented in the study are included in the article/Supplementary Material, further inquiries can be directed to the corresponding author.
