# Supplementary material for: Placebo-associated changes in MRI-PDFF and metabolic parameters in MASLD patients from phase Ib/IIa clinical trials: implications for early-phase trial design and translational endpoint interpretation
Source: Front Pharmacol. 2026 Mar 24;17:1782232. doi: 10.3389/fphar.2026.1782232 (PMC13055534; doi:10.3389/fphar.2026.1782232)
Supplement: Supplementary file 1 [file Supplementaryfile1.docx]

**Supplementary Material Table S1. Demographics and baseline characteristics of participants across the five parent trials.**

| **Characteristic** | **Total (41)** | **Trial 1 (12)** | **Trial 2 (7)** | **Trial 3 (7)** | **Trial 4 (9)** | **Trial 5 (6)** | **P Value** |
| --- | --- | --- | --- | --- | --- | --- | --- |
| Age, years | 41.0 (11.0) | 38.5 (10.7) | 36.9 (6.8) | 44.3 (12.3) | 49.6 (11.3) | 34.3 (6.8) | 0.1236 |
| Female | 16 (39) | 5 (42) | 3 (43) | 2 (29) | 4 (44) | 2 (33) | 0.992 |
| Male | 25 (61) | 7 (58) | 4 (57) | 5 (71) | 5 (56) | 4 (67) |  |
| Body weight, kg | 81.5 (14.0) | 91.9 (11.5) | 82.6 (6.6) | 71.7 (6.2) | 65.8 (7.1) | 94.2 (8.4) | <0.0001 |
| BMI, kg/m^2^ | 29.4 (4.5) | 32.5 (4.6) | 30.2 (3.8) | 26.2 (1.4) | 25.1 (2.4) | 32.6 (1.4) | 0.0002 |
| Type 2 Diabetes | 21 (41) | 3 (25) | 2 (14) | 7 (100) | 9 (100) | 0 (0) | <0.0001 |
| HbA1c, % | 7.4 (1.4) | 6.5 (0.5) | 6.5 (0.8) | 8.1 (0.9) | 8.9 (0.9) | 5.7 (0.5) | <0.0001 |
| FBG, mmol/L | 7.44 (2.42) | 6.06 (0.70) | 6.52 (1.05) | 9.16 (2.48) | 10.05 (2.37) | 5.35 (0.67) | <0.0001 |
| HOMA-IR | 5.3 (3.0) | NA | 6.8 (4.1) | 5.3 (2.6) | 5.4 (2.8) | 3.4 (1.6) | 0.4189 |
| ALT, U/L | 53.29 (35.91) | 71.28 (36.24) | 95.14 (23.69) | 23.14 (9.28) | 28.61 (14.70) | 40.68 (22.66) | <0.0001 |
| AST, U/L | 36.84 (25.29) | 51.76 (35.99) | 51.87 (13.19) | 18.99 (4.17) | 26.47 (14.43) | 25.85 (8.38) | 0.0002 |
| GGT, U/L | 73.80 (58.10) | 81.80 (48.07) | 97.56 (44.27) | 39.20 (19.88) | 87.06 (93.02) | 50.53 (40.82) | 0.0409 |
| ALP, U/L | 87.51 (21.15) | 98.52 (23.13) | 81.91 (22.79) | 85.54 (17.62) | 86.24 (22.09) | 76.23 (11.39) | 0.2869 |
| TC, mmol/L | 5.31 (0.86) | 5.30 (0.55) | 5.21 (1.11) | 5.03 (0.61) | 5.24 (1.18) | 5.87 (0.78) | 0.6423 |
| TG, mmol/L | 2.69 (1.13) | 2.74 (0.92) | 3.33 (1.27) | 2.25 (1.20) | 2.61 (1.43) | 2.48 (0.64) | 0.4675 |
| HDL, mmol/L | 1.16 (0.24) | 1.06 (0.13) | 1.13 (0.26) | 1.11 (0.18) | 1.28 (0.30) | 1.27 (0.28) | 0.5886 |
| LDL, mmol/L | 3.41 (0.67) | 3.42 (0.43) | 3.44 (0.84) | 3.20 (0.44) | 3.23 (0.96) | 3.84 (0.52) | 0.5876 |
| MRI-PDFF, % | 14.56 (8.60) | 18.47 (5.04) | 24.44 (11.67) | 8.13 (2.83) | 8.32 (3.10) | 12.08 (6.19) | 0.0002 |
| CAP, db/m | 314.5 (43.9) | 326.6 (41.9) | 338.4 (23.4) | 269.7 (30.7) | NA | NA | 0.0172 |
| LSM, kPa | 7.32 (2.97) | 8.73 (3.52) | 7.01 (1.84) | 5.19 (1.05) | NA | NA | 0.0414 |

Data are present as number (percent) or mean (standard deviation). Numbers in parentheses indicate the number of participants enrolled in each trial. NA, not available. BMI, body mass index; HbA1c, hemoglobin A1c; FBG, fasting blood glucose; HOMA-IR, homeostasis model assessment of insulin resistance; ALT, alanine aminotransferase; AST, aspartate aminotransferase; GGT, gamma-glutamyl transferase; ALP, alkaline phosphatase; TC, total cholesterol; TG, triglycerides; HDL, high-density lipoprotein cholesterol; LDL, low-density lipoprotein cholesterol; MRI-PDFF, magnetic resonance imaging–proton density fat fraction; CAP, controlled attenuation parameter; LSM, liver stiffness measurement.

**Supplementary Material Table S2.** **Baseline and follow-up demographic and clinical characteristics.**

| **Characteristic** | **Week 0 (18)** | **Week 4/5 (18)** | **Week 16 (18)** | **Week 56 (18)** |
| --- | --- | --- | --- | --- |
| Body weight, kg | 81.7 (12.0) | 80.0 (10.8) | 81.0 (11.4) | 81.3 (12.2) |
| BMI, kg/m^2^ | 29.1 (3.6) | 28.5 (3.2) | 28.9 (3.6) | 28.9 (3.6) |
| FBG, mmol/L | 7.25 (2.02) | 8.02 (4.43) | 7.16 (2.44) | 7.06 (1.84) |
| HOMA-IR | 4.7 (1.8) | 6.9 (3.5) | 5.8 (2.9) | 4.2 (2.3) |
| AST, U/L | 47.90 (39.10) | 38.94 (23.66) | 36.58 (16.05) | 38.10 (18.40) |
| ALT, U/L | 62.97 (39.51) | 54.81 (29.39) | 51.64 (28.48) | 58.29 (39.13) |
| GGT, U/L | 83.98 (64.44) | 72.02 (59.23) | 71.84 (48.86) | 75.44 (51.98) |
| ALP, U/L | 86.39 (18.23) | 84.55 (19.32) | 85.98 (13.37) | 85.64 (15.99) |
| TC, mmol/L | 5.26 (0.83) | 5.24 (0.72) | 5.37 (1.00) | 5.53 (0.58) |
| TG, mmol/L | 3.13 (1.23) | 3.12 (2.16) | 2.79 (1.30) | 3.21 (2.50) |
| LDL, mmol/L | 3.34 (0.70) | 3.30 (0.73) | 3.40 (0.65) | 3.48 (0.51) |
| HDL, mmol/L | 1.07 (0.21) | 1.07 (0.17) | 1.11 (0.19) | 1.69 (2.48) |
| MRI-PDFF, % | 17.68 (9.67) | 14.96 (8.36) | 15.80 (8.47) | 16.79 (8.54) |
| CAP, db/m | 327.6 (36.8) | 321.1 (47.4) | 328.8 (42.8) | 319.7 (47.0) |
| LSM, kPa | 7.15 (2.66) | 7.03 (1.87) | 6.81 (1.69) | 6.96 (2.14) |

Note. Week 0 refers to the baseline prior to placebo treatment; Week 4/5 indicates the end of the 4- or 5-week clinical trials; Week 16 and Week 56 correspond to the 3-month and 1-year follow-up after trial completion, respectively. BMI, body mass index. Numbers in parentheses indicate the number of participants with available data at each time point. Data are expressed as mean (standard deviation).

**Supplementary Material Table S3. Absolute changes during one-year follow-up in the five parent trials.**

| **Variables** | **Δ (Week 4/5 – Week 0)** | **Δ (Week 16 – Week 0)** | **Δ (Week 56 –Week 0)** | **Δ (Week 16 – Week 4/5)** | **Δ (Week 56 – Week 4/5)** |
| --- | --- | --- | --- | --- | --- |
| Body weight, kg | –1.7 (1.5) | –0.7 (1.5) | –0.4 (2.3) | 1.0 (1.2) | 1.3 (3.0) |
| BMI, kg/m^2^ | –0.6 (0.6) | –0.2 (0.5) | –0.1 (0.8) | 0.4 (0.5) | 0.5 (1.1) |
| FBG, mmol/L | 0.77 (3.10) | –0.09 (1.52) | –0.20 (1.27) | –0.86 (2.30) | –0.97 (3.43) |
| HOMA-IR | 2.2 (2.1) | 1.1 (3.1) | –0.5 (2.8) | –1.1 (3.4) | –2.7 (4.0) |
| AST, U/L | –8.96 (18.96) | –11.32 (30.20) | –9.80 (29.33) | –2.35 (16.18) | –0.84 (19.36) |
| ALT, U/L | –8.16 (19.59) | –11.33 (25.43) | –4.68 (28.98) | –3.17 (11.73) | 3.48 (24.41) |
| GGT, U/L | –11.96 (13.94) | –12.14 (31.14) | –8.54 (35.19) | –0.18 (30.02) | 3.42 (38.22) |
| ALP, U/L | –1.84 (14.76) | –0.41 (14.33) | –0.75 (15.36) | 1.43 (14.89) | 1.09 (15.13) |
| TC, mmol/L | –0.02 (0.66) | 0.11 (0.60) | 0.27 (0.72) | 0.13 (0.56) | 0.29 (0.71) |
| TG, mmol/L | –0.01 (2.18) | –0.34 (1.14) | 0.08 (2.52) | –0.33 (1.94) | 0.09 (2.19) |
| LDL, mmol/L | –0.04 (0.45) | 0.05 (0.43) | 0.14 (0.52) | 0.09 (0.44) | 0.18 (0.58) |
| HDL, mmol/L | 0.00 (0.18) | 0.04 (0.20) | 0.62 (2.52) | 0.04 (0.19) | 0.62 (2.50) |
| MRI-PDFF, % | –2.72 (3.04) | –1.88 (2.46) | –0.89 (5.89) | 0.84 (1.84) | 1.83 (5.93) |
| CAP, db/m | –6.5(34.8) | 1.1 (20.9) | –7.9 (32.0) | 7.6 (34.8) | –1.4 (47.1) |
| LSM, kPa | –0.12 (2.93) | –0.34 (1.87) | –0.18 (1.73) | –0.23 (2.25) | –0.07 (2.52) |

Note. Δ represents absolute changes in body weight, metabolic parameters, imaging parameters between two time points. Week 0, baseline; Week 4/5, end of the 4- or 5-week clinical trial; Week 16, 3-month follow-up; Week 56, 1-year follow-up. Δ (Week 4/5 – Week 0), change from baseline to end of placebo treatment; Δ (Week 16 – Week 0), change from baseline to 3-month follow-up; Δ (Week 56 – Week 0), change from baseline to 1-year follow-up; Δ (Week 16 – Week 4/5), change from end of placebo treatment to 3-month follow-up; Δ (Week 56 – Week 4/5), change from end of placebo treatment to 1-year follow-up. Data are expressed as mean change (standard deviation).

**Supplementary Material Table S4. Relative changes during one-year follow-up in the five parent trials.**

| **Variables (%)** | **Δ (Week 4/5 – Week 0)** | **Δ (Week 16 – Week 0)** | **Δ (Week 56 – Week 0)** | **Δ (Week 16 – Week 4/5)** | **Δ (Week 56 – Week 4/5)** |
| --- | --- | --- | --- | --- | --- |
| Body weight | –1.93 (1.74) | –0.79 (1.70) | –0.53 (2.72) | 1.18 (1.58) | 1.48 (3.74) |
| BMI | –1.88 (1.74) | –0.76 (1.73) | –0.51 (2.76) | 1.16 (1.60) | 1.45 (3.77) |
| FBG | 6.77 (33.16) | –1.00 (17.11) | –0.92 (15.52) | –4.04 (16.05) | –1.62 (24.07) |
| HOMA-IR | 43.48 (39.32) | 36.77 (82.91) | –1.43 (61.46) | –5.30 (46.73) | –22.35 (61.08) |
| AST | –6.05 (27.92) | –5.00 (36.06) | –3.64 (35.14) | 0.97 (24.53) | 6.09 (38.46) |
| ALT | –4.10 (26.48) | –3.99 (42.70) | 3.74 (49.79) | –0.73 (30.85) | 11.00 (48.09) |
| GGT | –13.32 (16.33) | –5.89 (25.99) | –4.68 (31.42) | 9.15 (24.18) | 14.30 (46.38) |
| ALP | –0.93 (17.42) | 2.04 (18.17) | 1.19 (17.70) | 4.31 (16.90) | 3.75 (19.43) |
| TC | 0.72 (13.34) | 2.29 (10.54) | 6.73 (15.11) | 2.37 (10.17) | 6.74 (14.04) |
| TG | 10.59 (88.11) | –7.07 (34.85) | 9.70 (90.04) | 5.00 (39.47) | 16.18 (73.95) |
| LDL | –0.44 (15.78) | 2.78 (13.04) | 6.44 (16.62) | 5.46 (21.35) | 9.66 (27.19) |
| HDL | 2.20 (17.73) | 5.64 (18.37) | 69.74 (273.10) | 4.43 (16.59) | 64.47 (255.00) |
| MRI-PDFF | –11.63 (25.44) | –8.45 (17.72) | –1.58 (35.50) | 6.66 (15.95) | 18.91 (54.53) |
| CAP | –1.94 (10.86) | 0.30 (6.20) | –2.37 (10.20) | 3.25 (11.06) | 0.83(16.56) |
| LSM | 6.43 (40.50) | –0.55 (23.14) | –0.10 (19.91) | 1.31 (27.73) | 3.15 (32.91) |

Note. Δ represents relative changes percent in body weight, metabolic parameters, imaging parameters between two time points. Week 0, baseline; Week 4/5, end of the 4- or 5-week clinical trial; Week 16, 3-month follow-up; Week 56, 1-year follow-up. Δ (Week 4/5 – Week 0), change from baseline to end of placebo treatment; Δ (Week 16 – Week 0), change from baseline to 3-month follow-up; Δ (Week 56 – Week 0), change from baseline to 1-year follow-up; Δ (Week 16 – Week 4/5), change from end of placebo treatment to 3-month follow-up; Δ (Week 56 – Week 4/5), change from end of placebo treatment to 1-year follow-up. Data are expressed as relative mean change percent (standard deviation).

**Supplementary Material Figure S1. Changes in hepatic fat content in participants from five clinical trials.**


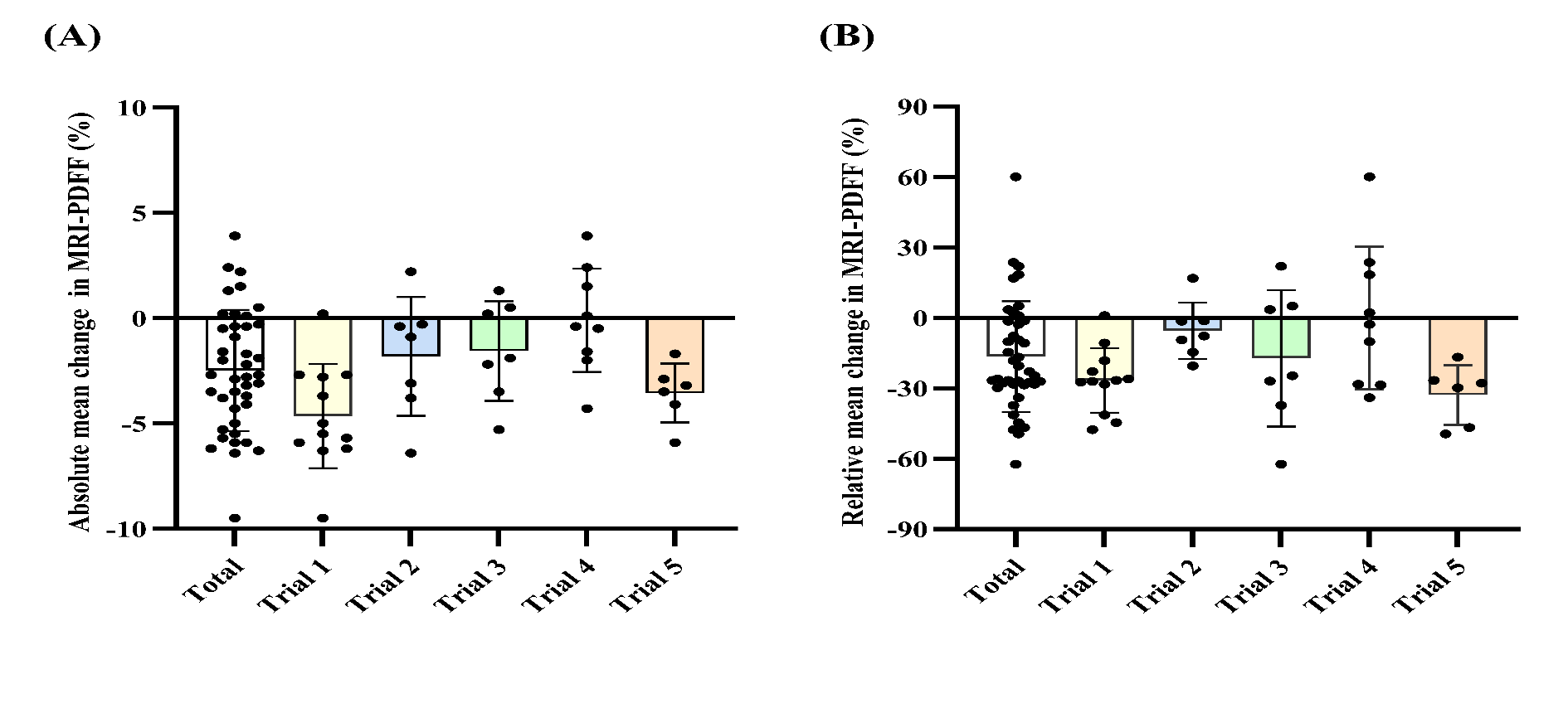


(A) Absolute mean change from baseline to the end of placebo treatment in MRI-PDFF for each of the five phase Ib/IIa clinical trials. (B) Relative mean change from baseline to the end of placebo treatment in MRI-PDFF for each of the five clinical trials. Bars represent mean values, with error bars indicating standard deviation (SD). Each black dot represents an individual participant.

**Supplementary Material Figure S2. Proportion of participants (N = 18) with ≥30% change (increase or decrease) in hepatic fat content and ≥5% change (loss or gain) in body weight over one year.**


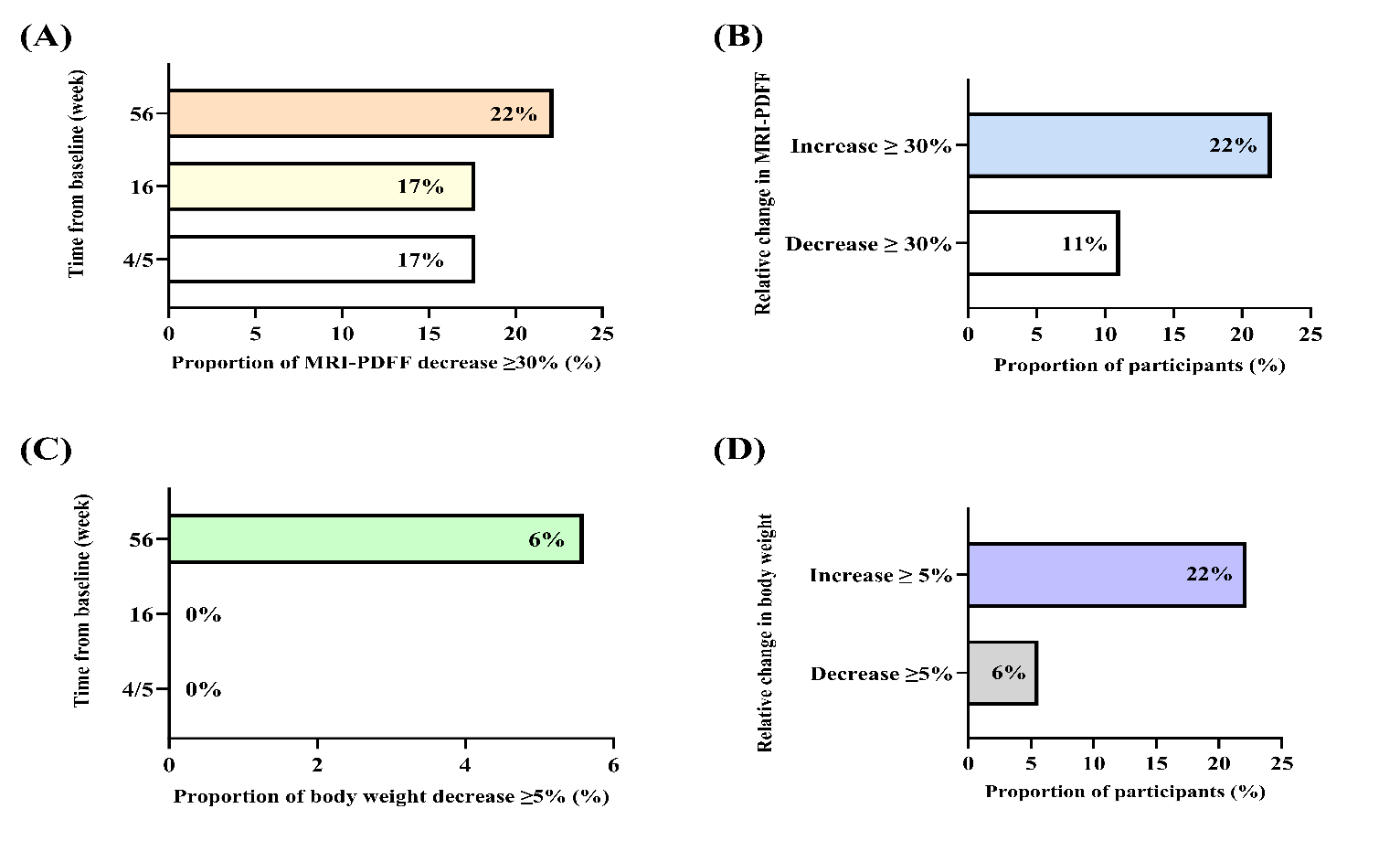


(A, C) Proportion with ≥30% decrease in MRI-PDFF (A) and ≥5% body weight loss (C) at week 4/5 (end of trial treatment), week 16 (3-month follow-up), and week 56 (1-year follow-up), compared to baseline (week 0), respectively; (B) Proportion with ≥30% relative change (increase or decrease) in MRI-PDFF from week 4/5 to week 56; (D) Proportion with ≥5% relative change (increase or decrease) in body weight from week 4/5 to week 56.

**Supplementary Material Figure S3. Absolute mean and absolute mean changes in lipid parameters among time points.**


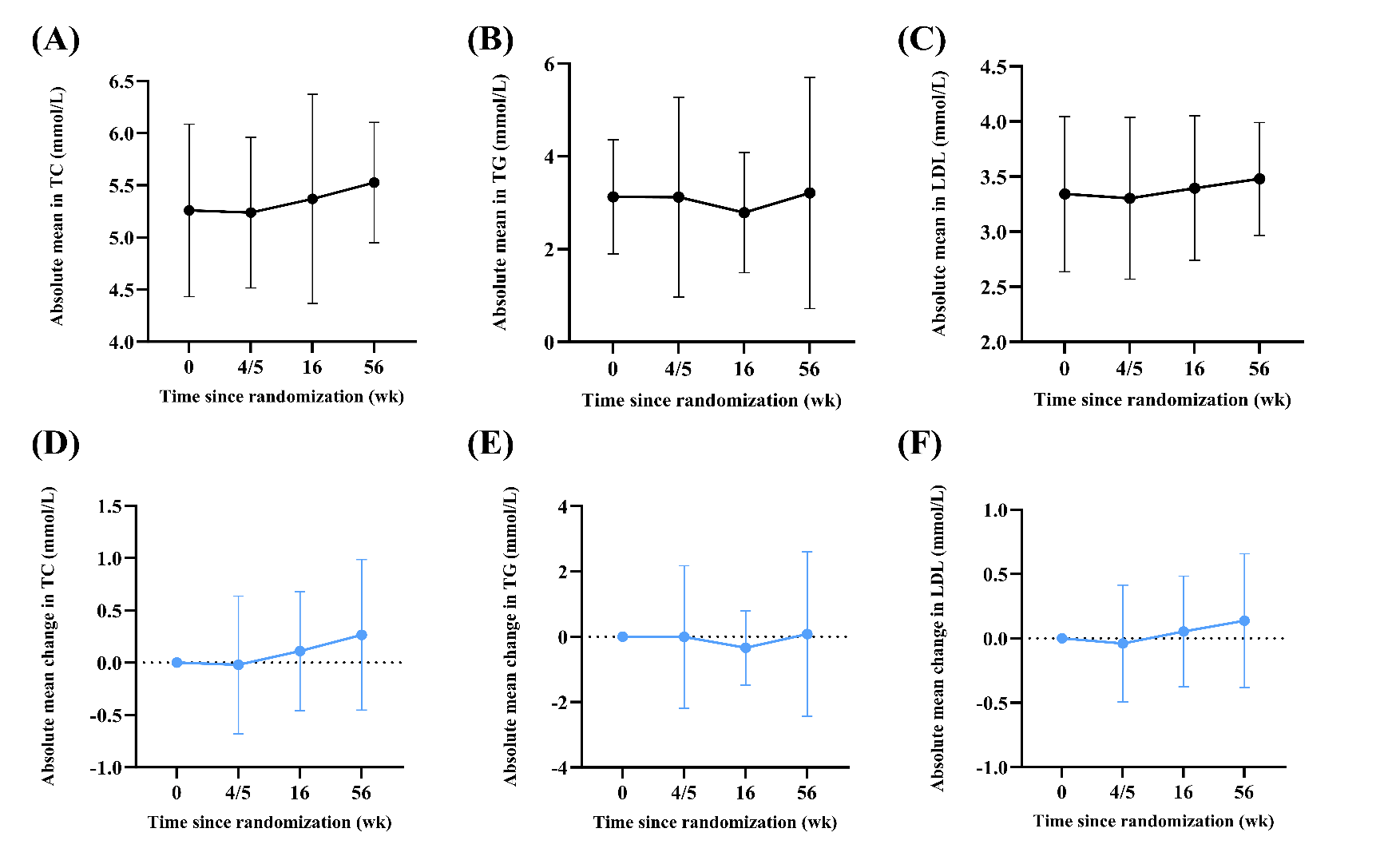


(A–C) Absolute mean in TC (A), TG (B) and LDL (C) at week 0, week 4/5, week 16 and week 56; (D–F) Absolute mean change in TC (D), TG (E) and LDL (F) at week 4/5, week 16, and week 56 compared to baseline. Data are plotted as mean or mean change ± standard deviation.
